# SG-APSIC1207: Effectiveness of a surgical-site infection bundle in reducing postoperative infection in cesarean deliveries in a tertiary-care teaching hospital in Malaysia

**DOI:** 10.1017/ash.2023.88

**Published:** 2023-03-16

**Authors:** Sasheela Sri La Sri Ponnampalavanar, Arulvani Rajandra, Nur Alwani Suhaimi, Cindy Teh Shuan Ji, Sia Jia Xuen, Tan Shu Fan, Siti Zuhairah binti Mohd Razali, Kam Yit Yin, Zhi Xian Kong, Min Yi Lau, Yee Qing Lee, Siti Norintan Zainon, Anjanna Kukreja, Suzana Saaibon, Siti Shuhaida Shamsudin

**Affiliations:** University Malaya Medical Centre, Malaysia

## Abstract

**Objectives:** Surgical-site infections (SSIs) cause significant increases in mortality, morbidity, and prolonged hospitalization after cesarean deliveries. We assessed the effectiveness of the implementation of an SSI bundle in reducing postoperative infections in cesarean deliveries in a tertiary-care teaching hospital in Malaysia. **Method:** We conducted a quality-improvement study on all women who underwent labor and scheduled cesarean sections at the University Malaya Medical Center (UMMC) between May and December 2020. The preintervention period was May–June 2020 and the postintervention period was September–December 2020. Patients were followed for 90 days after their operation. Before the intervention, SSI rates and compliance with prevention practices were documented. A multidisciplinary team was formed, and education regarding the elements of the SSI prevention bundle was conducted before they were implemented. The care bundle focused on monitoring compliance with preoperative bathing, contact time for skin preparation, hair management, and antibiotics prophylaxis given within 60 minutes prior to incision, as well as patient education. **Result:** With the implementation of the SSI bundle, we observed a significant reduction in the SSI rate by 50%, from 7 per 100 procedures to 2 per 100 procedures. Compared with the preintervention period, overall compliance with bundle elements improved greatly for preoperative bathing (0 vs 95.7%) and contact time for skin preparation (0 vs 98.8%). In the postintervention period, the method of hair removal was documented, compared to no documentation during the preintervention period. The administration of prophylactic antibiotics within 60 minutes prior incision decreased from 99% to 92.3%. **Conclusion:** Implementation of an SSI prevention bundle successfully reduced the rate of SSI after cesarean section. The SSI prevention bundle together with improvements by multidisciplinary teams and a good patient-safety culture helped reduce SSI rates. Patient education on pre- and postoperative infection prevention also played an important role in reducing this infection rate.

